# Pivotal Roles of Monocytes/Macrophages in Stroke

**DOI:** 10.1155/2013/759103

**Published:** 2013-01-27

**Authors:** Tsuyoshi Chiba, Keizo Umegaki

**Affiliations:** Information Center, National Institute of Health and Nutrition, 1-23-1 Toyama, Shinjuku-ku, Tokyo 162-8636, Japan

## Abstract

Stroke is an important issue in public health due to its high rates both of morbidity and mortality, and high rate of disability. Hypertension, cardiovascular disease, arterial fibrillation, diabetes mellitus, smoking, and alcohol abuse are all risk factors for stroke. Clinical observations suggest that inflammation is also a direct risk factor for stroke. Patients with stroke have high levels of inflammatory cytokines in plasma, and immune cells, such as macrophages and T-lymphocytes, are noted within stroke lesions. These inflammatory events are considered as a result of stroke. However, recent studies show that plasma levels of inflammatory cytokines or soluble adhesion molecules are high in patients without stroke, and anti-inflammatory therapy is effective at reducing stroke incidence in not only animal models, but in humans as well. Statins have been shown to decrease the stroke incidence via anti-inflammatory effects that are both dependent and independent of their cholesterol-lowering effects. These reports suggest that inflammation might directly affect the onset of stroke. Microglial cells and blood-derived monocytes/macrophages play important roles in inflammation in both onset and aggravation of stroke lesions. We review the recent findings regarding the role of monocytes/macrophages in stroke.

## 1. Introduction

Stroke is the third leading cause of death and a major cause of disability in industrialized countries. Ischemic stroke is the most common type of stroke, occurring in approximately 80% of all strokes [1]. A less common type of stroke is hemorrhagic stroke, which occurs due to a subarachnoid hemorrhage and/or an intracerebral hemorrhage. Hypertension, cardiovascular disease, arterial fibrillation, diabetes mellitus, obesity, smoking, and alcohol abuse are risk factors for stroke [2], even if there are slight differences in the influence of these factors between ischemic stroke and hemorrhagic stroke. However, some stroke patients do not have any of these risk factors, suggesting that other risk factors exist. For many years, clinical observations showed that plasma levels of inflammatory cytokines were increased after stroke onset, and immune cells, especially monocytes/macrophages and T-lymphocytes, existed in stroke lesions and related to exaggerate brain damage. In the clinical setting, elevated plasma levels of inflammatory cytokines, C-reactive protein (CRP), and chemokines are associated with future cardiovascular risk [3]. Plasma levels of soluble intercellular adhesion molecule-1 (sICAM-1) and sE-selectin were observed to be increased both in large intracranial artery disease and small-artery disease [4], and plasma levels of ICAM-1 and monocyte chemoattractant protein-1 (MCP-1) were noted to be high in patients with ischemic stroke and myocardial infarction [5, 6]. Epidemiological studies have shown that elevated leukocyte count was associated with the risk for first-time myocardial infarction and ischemic stroke [7–9] and the risk of recurrent myocardial infarction and ischemic stroke in a high-risk population [10]. These observations indicate that inflammatory events occur in stroke patients and increase the risk of stroke recurrence. Recently, both clinical and animal studies revealed that these inflammatory events occurred prior to stroke onset. Plasma levels of soluble vascular cell adhesion molecule-1 (sVCAM-1), sICAM-1, sE-selectin, and MCP-1 were elevated in patients with essential hypertension in the absence of other diseases [11–13]. Anti-inflammatory strategies were shown to suppress the incidence of stroke in both human and animal models. These reports suggest that inflammation might be a risk factor for stroke. We review the recent findings regarding the role of inflammation, especially monocytes/macrophages, in ischemic stroke which is predominant type of strokes.

## 2. Monocytes/Macrophages and Stroke

### 2.1. Atherosclerosis

Atherosclerosis is one of the major risk factors for stroke, and monocytes/macrophages affect the brain indirectly by inducing unstable plaques and plaque rupture in atherosclerotic lesions [14]. It is well recognized that atherosclerosis is an inflammatory disease and macrophages play important roles in the initiation and the progression of atherosclerotic lesion [15]. Accumulation of monocytes/macrophages in the vascular wall occurs early during atherosclerosis [15]. In addition to phagocytosis of oxidized low-density lipoproteins, macrophages secrete interleukin-1*β* (IL-1*β*), tumor necrosis factor-*α* (TNF-*α*), and transforming growth factor-*β*1 (TGF-*β*1). These inflammatory cytokines and growth factors induce endothelial dysfunction, smooth muscle cell migration and proliferation, and extracellular matrix production as fibrous plaques. During later disease stages, activated macrophages secrete several classes of neutral extracellular proteases, including serine proteases, cathepsins, and matrix metalloproteinases (MMPs) [16]. Blood monocytes already express low levels of a few MMPs [17]; however, contact with matrix leads to rapid upregulation of a broad spectrum of MMPs. Cell biology experiments identify mechanisms by which excessive MMP production can cause plaque rupture, either directly by destruction of extracellular matrix [18] or indirectly through actions that promote death of macrophages [19] and vascular smooth muscle cells [20]. Rupture of unstable plaques may lead to thrombotic stroke onset.

### 2.2. At the Brain

Monocytes/macrophages directly play important roles in stroke at the brain. Microglial cells, the resident macrophages of the brain, and blood-derived monocytes/macrophages have morphologically and functionally similar roles in stroke [21, 22]. Microglial cells are activated rapidly in response to brain injury [23]. This activation occurs within minutes of ischemia onset and induces production of inflammatory cytokines, including IL-1*β* and TNF-*α*, which exacerbate tissue damage [24–26]. Following the rapid activation of resident microglial cells, blood-derived immune cells infiltrate into the brain tissue within hours to a few days [21, 22]. Most current data from mice models and humans show that blood-derived macrophages are recruited into the ischemic brain tissue, most abundantly at days 3 to 7 after stroke [27–29]. In contrast, resident microglial cells are already activated rapidly on day 1 after focal cerebral ischemia. Resident microglial cells exist in lesions even at days 4 through 7. These reports suggest that the resident microglial cell activation is induced immediately after brain injury and then blood-derived macrophage infiltration follows. On the other hand, it is reported that macrophages exist in the brain before onset of stroke in stroke-prone spontaneously hypertensive rats (SHRSP) [30, 31]. These findings suggest that the alteration of the blood-brain barrier and macrophage activation occurs before the onset of stroke, and these changes might induce stroke onset.

### 2.3. Activation of Immune Cells

Neutrophils and lymphocytes are also observed in stroke lesions. In ischemic stroke mice model, macrophages started to appear already at 12 hours after ischemia. On the other hand, lymphocytes (B- and T-lymphocytes) and neutrophils were significantly increased at 3 days after ischemia [32]. According to this observation, it was reported that macrophages produce inflammatory cytokines and upregulate adhesion molecules in endothelial cells, thereby promoting neutrophil accumulation and migration into the brain [33]. These data suggest that macrophage infiltration occurs prior to other immune cells and macrophage activation attracts other immune cells into stroke lesions. Different subtypes of T-lymphocytes play differential roles in the stroke. CD4^+^ TH1 cells may progress stroke through releasing proinflammatory cytokines, including IL-2, IL-12, IFN-*γ*, and TNF-*α*, whereas CD4^+^ TH2 cells may play a protective role through releasing anti-inflammatory cytokines such as IL-4, IL-5, IL-10, and IL-13 [34]. However, exact role of neutrophils in the stroke is still unclear.

## 3. Relationship between Monocytes/Macrophages and Hypertension

Hypertension is the principal risk factor for stroke and is a leading cause of cognitive decline and dementia [35]. There is a linear relationship between blood pressure and stroke mortality [36]. Hypertension might induce endothelial cell dysfunction along with macrophage activation and infiltration into the brain. There is emerging evidence that monocyte/macrophage infiltration contributes to hypertension [37].

### 3.1. Endothelial Cell Dysfunction

Endothelial cell dysfunction is the first step of monocytes/macrophages infiltration into brain. Hypertension might induce endothelial cell dysfunction [38], vascular inflammation on the vascular lumen [39], and monocyte adhesion [40]. It was reported that hypertension promoted or aggravated endothelial dysfunction, which induced the expression of ICAM-1, P-selectin, and monocyte adhesion in a rat model [40]. High intraluminal pressure activated NF*κ*B in an organ culture model of mouse carotid arteries [41]. In humans, the association of chronically or acutely elevated blood pressure with markers of inflammation has also been documented. Circulating levels of sICAM-1, sVCAM-1, sE-selectin, and MCP-1 are increased in patients with essential hypertension [13, 42]. Increasing levels of adhesion molecules and chemoattractant molecules could induce monocyte adhesion on the vascular surface and migration into subendothelial lesions in both aortae and the brain.

### 3.2. Monocyte/Macrophage Activation

Hypertension might affect blood monocytes directly. The total number of blood monocytes and activated monocytes is greater in spontaneously hypertensive rats compared with Wistar Kyoto rats, which represent the normotensive control [43, 44]. On the other hand, reducing blood pressure with angiotensin converting enzyme inhibitors suppresses endothelial dysfunction and the number of subendothelial macrophages in the aorta [45]. In humans, circulating monocytes from patients with essential hypertension are preactivated compared with those in normotensive healthy individuals. IL-1*β* secretion of peripheral blood monocytes stimulated by angiotensin II was shown to be significantly higher in patients with essential hypertension compared with normotensive healthy individuals [46].

### 3.3. Renal Dysfunction

Inflammatory cells accumulate in perivascular regions in the kidney, and in and around glomeruli in hypertensive rats [47, 48] and hypertensive subjects [49]. There is extensive perivascular infiltration of leukocytes in the kidney of double transgenic rats harboring human renin and angiotensinogen genes. In a study that emphasized the role of inflammation in blood pressure elevation, pyrrolidine dithiocarbamate, an inhibitor of NF*κ*B, prevented monocyte/macrophage infiltration in animals, reduced expression of ICAM-1 and inducible nitric oxide synthase, and reduced blood pressure [48]. There is also evidence of macrophage infiltration in the glomeruli of hypertensive animals [50] and humans [49]. Monocytes/macrophages in the kidney modulate blood pressure via the production of inflammatory cytokines and modulation of renin-angiotensin-aldosterone system [51, 52]. On the other hand, drugs acting on the renin-angiotensin-aldosterone system prevent or modulate inflammation [53]. Monocytes/macrophages might play some important roles in the reciprocal influence between inflammation and hypertension.

## 4. Animal Models

### 4.1. Stroke-Prone Spontaneously Hypertensive Rats

SHRSPs are unique genetic model that mimic both microvessel and parenchymal changes in spontaneous stroke [54, 55]. The microvascular changes and brain parenchymal damage may not simply be the result of hypertension, and endothelial cell dysfunction [56] and inflammation may play a role in brain damage [55]. This animal model has been used to examine the contributions of inflammation (macrophages) to stroke. In SHRSP, fed a high-salt diet, rosuvastatin treatment significantly delayed the onset of stroke and attenuated the transcription of inflammatory biomarkers (MCP-1, TGF-*β*1, IL-1*β*, and TNF-*α*) [57]. Pioglitazone, peroxisome proliferator-activated receptor-*γ* agonist, reduced the risk of recurrent stroke in patients with type 2 diabetes [58]. In SHRSP, pioglitazone delayed the onset of stroke by improving vascular endothelial dysfunction, inhibiting brain inflammation, and reducing oxidative stress [59]. A low dose of acetylsalicylic acid (aspirin) delayed the onset of stroke in SHRSP by suppressing inflammation [60]. In addition to drug treatments, dietary restriction has been shown to delay the onset of stroke in SHRSP via suppression of systemic and local inflammation including macrophage infiltration into the brain [31].

### 4.2. Middle Cerebral Artery Occlusion

Permanent or transient middle cerebral artery occlusion is an established method for inducing focal ischemic stroke in mice or rats. Middle cerebral artery occlusion produces highly reproducible lesions, and macrophages primarily infiltrate into the core of the ischemic lesion [61]. The focal ischemic stroke model is a closer approximation to human stroke and produces a heterogeneous pathology that includes a necrotic core and salvageable penumbra [62]. However, small differences in surgical technique may account for different effects on the infarct [63, 64]. Furthermore, due to variances in cerebral vascular anatomy, different mouse strains show a different outcome [65, 66]. In addition, conditions of animals during surgery, such as blood pressure, blood gases, body temperature, and anesthesia influence outcome. Thus, standardization and quality control are very important when using this animal model.

### 4.3. Hypertensive Mice with Salt Loading

There are a lot of hypertensive animal models [67]; however, surgical intervention is needed to induce stroke in these models. Recently, these hypertensive mice have been used to research spontaneous stroke. Excessive salt intake induced frequent thoracic or abdominal cavity hemorrhage in Tsukuba hypertensive mice, which are human renin and angiotensinogen transgenic mice [68]. Hemorrhaging occurred due to the development of aortic aneurysms and rupture at the aortic arch and aorta near the renal arteries. Vascular lesions progressed with structural degeneration of the aortic media. Unfortunately, cerebral pathology was not assessed in this model [68]. Subsequently, a spontaneous stroke model using human renin and angiotensinogen transgenic hypertensive mice, but not Tsukuba hypertensive mice, was reported [69]. In this report, high-salt diet and L-NAME diet induced hemorrhage in the brain stem, cerebellum, and basal ganglia, which were reasonably similar to those observed in patients with hypertension. It is not clarified whether these mice models show ischemic stroke; however, these hypertensive mice, especially renin and angiotensinogen transgenic mice, are useful for experimental stroke research.

## 5. Inflammatory Cytokines

Inflammatory cytokines, such as IL-1*β*, IL-6, and TNF-*α*, are secreted by activated microglial cells and macrophages in stroke lesions and induce the expression of chemokines, which recruit more circulating monocytes/macrophages into lesions and lead to further brain damage. However, the role of each cytokine in stroke is complicated.

### 5.1. Interleukin-1*β*

Recently, IL-1*β* has been considered a therapeutic target for stroke. Chronic increases in IL-1*β* expression in the brain led to leukocyte infiltration and increased MCP-1 and ICAM-1 expressions in a mouse model [70], which is a phenotype also seen in stroke lesions. In addition, a number of studies have demonstrated that inhibiting the release or action of IL-1 markedly reduced ischemic cerebral damage in animal models. IL-1*α* and IL-1*β* double knockout mice exhibited dramatically reduced ischemic infarct volume compared with wild-type mice [71]. In a meta-analysis of animal model studies, IL-1 receptor antagonist (IL-1Ra), which represents the most advanced approach to modify IL-1 action, reduced infarct volume in models of focal cerebral ischemia [72]. In humans, a phase II clinical trial of intravenous IL-1Ra compared with placebo in patients with acute stroke is currently underway [73]. Further, IL-1Ra gene polymorphism represents a risk factor for ischemic stroke [74, 75]. These reports suggest that inhibition of IL-1*β* signals can prevent the onset of stroke.

### 5.2. Interleukin-6

A prospective cohort study and systemic review revealed that plasma levels of IL-6 were associated with poor outcome after both ischemic and hemorrhagic strokes [76]; however, it was not clear whether IL-6 increased before or after stroke onset. Animal models showed less association between IL-6 and stroke. IL-6 could not induce adhesion molecules and MCP-1 mRNA expressions in cerebrovascular endothelial cells derived from SHRSP [31]. Mice deficient in IL-6 showed similar stroke lesion volume and neurological function as control mice in an acute ischemic injury model [77]. Furthermore, IL-6 mediates anti-inflammatory effects in addition to its proinflammatory role [78]. Therefore, its manipulation can have either detrimental or beneficial effects. Further studies are required to clarify the role of IL-6 in stroke.

### 5.3. Tumor Necrosis Factor-*α*

Increased serum and cerebrospinal fluid levels of TNF-*α* have been found in patients 24 hours, 1 week, and 2 weeks after stroke, and these increases correlate with infarct volume and severity of neurological impairment [79]. However, previous reports suggest that TNF-*α* has a dual role in brain injury [80, 81]. Blockade of TNF-*α* actions reduced infarct volume after permanent middle cerebral artery occlusion in BALB/C mice with the dimeric type I soluble TNF receptor, which binds to TNF-*α* and antagonizes its action [82]. In contrast, TNF-*α* pretreatment was neuroprotective against permanent middle cerebral artery occlusion in BALB/C mice with reduction of infarct size, macrophages, and CD11b-positive neutrophils [83]. In addition to these observations, pentoxifylline, an anti-inflammatory agent, attenuated damage of stroke via the dual role of TNF-*α*. Pentoxifylline treatment increased serum levels of TNF-*α*, but not IL-1*β* and IL-6, and dose dependently prevented the occurrence of spontaneous brain damage by reducing macrophage infiltration into lesion in SHRSP [84], suggesting a protective role of TNF-*α*. On the other hand, pentoxifylline reduced brain edema in a rat model of transient focal cerebral ischemia through a decline in TNF-*α* production [85], suggesting an deleterious role of TNF-*α*. Although anti-TNF-*α* strategies have proved beneficial in other clinical settings such as inflammatory bowel disease, there are no clinical trials of anti-TNF-*α* agents in stroke. Further studies are required to clarify the role of TNF-*α* in stroke.

### 5.4. MCP-1

CC chemokine ligand (CCL2) is known as MCP-1 and is a potent mononuclear cell attractant. MCP-1 is synthesized by several cell types, such as monocytes/macrophages, T-lymphocytes, smooth muscle cells, endothelial cells, and even cerebrovascular endothelial cells. Expression of MCP-1 is upregulated by inflammatory cytokines. Serum levels of MCP-1 are high in patients with ischemic stroke and myocardial infarction [5, 6], which might be interpreted as a stroke-induced increases in inflammatory events. On the other hand, there is one report that serum CCL2 levels in acute ischemic stroke patients did not differ from that in controls at 1 to 3 days after stroke onset [86]. In this paper, details of controls were not shown, but one of the possibilities is that control subjects were hypertensive. It is reported that plasma levels of MCP-1 were elevated in patients with essential hypertension in the absence of other diseases [13]. The MCP-1-deficient mice model is a unique model to elucidate the role of macrophages in stroke [87]. Compared with control mice, infarct volume was smaller in MCP-1-deficient mice 24 hours after middle cerebral artery occlusion, and a reduction of phagocytic macrophage accumulation within infarcts and the infarct border in MCP-1 deficient mice 2 weeks after middle cerebral artery occlusion. In addition, MCP-1 deficient mice produced less IL-1*β* in ischemic tissue. This means that MCP-1 and IL-1*β* are key factors of macrophages in stroke lesions.

### 5.5. Adipokines

Obesity is also recognized as the risk factor for stroke, because obesity is associated with hypertension and inflammation via secretion of adipokines, such as adiponectin, leptin, resistin, adipsin, plasminogen activator inhibitor-1, and inflammatory cytokines [88–90]. It is well known that macrophage infiltration into adipose tissue induces inflammation in adipose tissue and influences these adipokine secretions [91, 92]. The most commonly studied adipocytokines are leptin and adiponectin. There are a lot of reports about the association of leptin and adiponectin with stroke, and leptin and adiponectin show differential association patterns with ischemic stroke [93]. It is reported that higher leptin levels and lower adiponectin levels were found in stroke patients [94]. On the other hand, there are controversial reports that adiponectin, but not leptin, levels are recognized as a predictor of the risk for stroke [95], or that leptin, but not adiponectin, levels are recognized as a predictor of the risk for stroke in men, but not women [96]. It is not clear whether adiponectin and leptin are useful predictors of stroke in obese subjects; however, adiponectin and leptin might directly influence stroke incidence. It is reported that leptin stimulates macrophages and that adiponectin suppresses it. Leptin increases the mRNA and protein levels of IL-1*β*, IL-6, IL-12, TNF-*α*, cyclooxygenase-2, and MCP-1 in macrophages and endothelial cells [97, 98]. Adiponectin inhibits pro-inflammatory signaling in human macrophages [99] and promotes macrophage polarization toward an anti-inflammatory phenotype [100]. Adiponectin also increases IL-10, an anti-inflammatory cytokine, as well as mRNA expression in human monocyte-derived macrophages [101]. In addition, both adiponectin and leptin receptors are expressed in the brain, suggesting that these adipokines might be directly associated with stroke [102, 103].

## 6. Anti-Inflammatory Strategies

There are several reports that treatment with drugs that have anti-inflammatory properties can prevent stroke not only in animal models, but also in humans.

### 6.1. Statins

Rosuvastatin treatment significantly delayed the onset of stroke and attenuated the transcription of inflammatory biomarkers [57]. Clinical studies using statins already use inflammatory events as endpoints for stroke prevention. In healthy persons without hyperlipidemia but with elevated high-sensitivity CRP levels, rosuvastatin, which lowered high-sensitivity CRP as well as cholesterol levels, reduced the incidence of stroke and myocardial infarction by 50% relative to placebo [104]. A meta-analysis of statin trials showed that statins might reduce the incidence of all strokes by decreasing LDL-cholesterol without increasing the incidence of hemorrhagic stroke [105]. In addition to cholesterol-dependent effects, cholesterol-independent effects of statins on stroke have also been recognized [106, 107]. However, statin treatment increases the risk of hemorrhagic stroke in patients with a history of cerebrovascular disease, even though it also clearly decreased the risk of ischemic stroke [108]. Therefore, patients undergoing statin treatment should be carefully monitored to avoid achieving very low level of cholesterols, which are known risk for hemorrhagic stroke [109].

### 6.2. Thiazolizinediones

Thiazolidinediones, including rosiglitazone and pioglitazone, are peroxisome proliferator-activated receptor-*γ* (PPAR-*γ*) agonists used in the treatment of type 2 diabetes. A systemic review showed that rosiglitazone and pioglitazone were similarly effective in reducing infarct volume and protecting neurologic function in a rodent model of focal or global cerebral ischemia [110]. Pioglitazone delayed the onset of stroke by improving vascular endothelial dysfunction and brain inflammation in SHRSP. Pioglitazone suppressed macrophage infiltration, MCP-1 and TNF-*α* gene expressions in the brain [59]. Rosiglitazone induced upregulation of CD36 in macrophages and enhanced the ability of microglia to phagocytose red blood cells, which helped to improve hematoma resolution, and improved functional deficits in an intracerebral hemorrhage mouse model [111]. In humans, the PROspective pioglitAzone Clinical Trial In macroVascular Events (PROACTIVE) [112] showed that pioglitazone significantly reduced the risk of recurrent stroke in high-risk patients with type 2 diabetes [58]. On the other hand, one report showed that compared with pioglitazone, rosiglitazone was associated with an increased risk of stroke, heart failure, and all-cause mortality and an increased risk of the composite of acute myocardial infarction, stroke, heart failure, or all-cause mortality in patients of 65 years or older [113].

### 6.3. Other Anti-Inflammatory Drugs

Low-dose acetylsalicylic acid (aspirin) also delayed the onset of stroke in SHRSP via suppression of inflammation [60]. Aspirin reduced salt-induced macrophage accumulation and MMP-9 activity at the stroke-negative area in the cerebral cortex of SHRSP [60]. Frequent aspirin use might also confer a protective effect for risk of stroke in humans [114, 115]. Terutroban, a specific thromboxane/prostaglandin endoperoxide receptor antagonist, decreased cerebral mRNA expressions of IL-1*β*, transforming growth factor-*β*, and MCP-1 and increased survival in SHRSP [116]. These effects were similar to rosuvastatin and aspirin [116]. The Prevention of cerebrovascular and cardiovascular Events of ischemic origin with terutroban in patients with a history of ischemic stroke or transient ischemic attack (PERFORM) study was started in February 2006 [117]. Recently, it was reported that PERFORM study did not meet the predefined criteria for noninferiority, but showed similar rates to terutroban and aspirin for the primary endpoint, such as a composite of fatal or nonfatal ischemic stroke, fatal or nonfatal myocardial infarction, or other vascular death [118]. These reports indicate that antiplatelets agents, which also have anti-inflammatory properties, could suppress inflammation and prevent stroke onset.

## 7. Beneficial Roles after Stroke

It is generally believed that the activated microglial cells in ischemic injury are neurotoxic, and results of several recent studies revealed that microglial cells might exert neuroprotective effects in certain conditions [119, 120]. In addition to the primary role of macrophages, which is the phagocytosis of cellular and fibrillar debris resulting from stroke, activated microglial cells and macrophages are involved in regulation of the regenerative state and remodeling of the brain by producing brain-derived neurotrophic factor [121, 122], insulin growth factor 1 [123, 124], several other growth factors [125], neuroprotective gene Ym1 [126], and nitric oxide which are known to regulate synaptic functions [127]. As described previously, some cytokines secreted from microglial cells and macrophages, such as IL-6 and TNF-*α*, and attenuate brain damage. In addition to these mediators, intracranial transplantation of monocyte-derived multipotential cells enhances recovery after ischemic stroke [128]. Whether activated microglial cells and macrophages act as toxic or neuroprotective factors might depend on the time and severity of stroke lesions.

## 8. Summary

**Figure 1:**
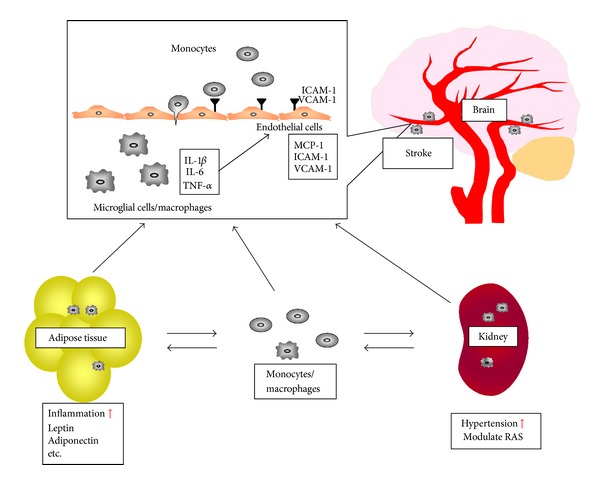
Monocytes/macrophages modulate adipose tissue and kidney functions and accelerate stroke. Monocytes/macrophages infiltration into adipose tissue stimulates secretion of leptin and inflammatory cytokines and suppresses secretion of adiponectin, which induce systemic inflammation, endothelial cell dysfunction, and monocytes/macrophages activation. Monocytes/macrophages infiltration into kidney modulates renin-angiotensin system and increase blood pressure, which also induces endothelial cell dysfunction and monocytes/macrophages activation. Endothelial cells express MCP-1 and adhesion molecules, which induce monocytes chemotaxis, adhesion, and migration into subendothelial lesions. Microglial cells and infiltrated monocytes/macrophages in brain induce cerebrovascular damages and cause stroke onset.

Microglial cells and monocytes/macrophages play important roles in the onset and aggravation of stroke via expression of several inflammatory cytokines at the brain, adipose tissue, and kidney (Figure 1). However, it is also reported that these inflammatory events are important in the reduction of and recovery from brain damage. However, it is clear that suppression of inflammation is effective in the prevention of primary stroke, and macrophages might be therapeutic targets to prevent stroke.
